# How Much Is Too Little to Detect Impacts? A Case Study of a Nuclear Power Plant

**DOI:** 10.1371/journal.pone.0047871

**Published:** 2012-10-26

**Authors:** Mariana Mayer-Pinto, Barbara L. Ignacio, Maria T. M. Széchy, Mariana S. Viana, Maria P. Curbelo-Fernandez, Helena P. Lavrado, Andrea O. R. Junqueira, Eduardo Vilanova, Sérgio H. G. Silva

**Affiliations:** Universidade Federal do Rio de Janeiro, Instituto de Biologia, Rio de Janeiro, Rio de Janeiro, Brazil; Université du Québec à Rimouski Canada

## Abstract

Several approaches have been proposed to assess impacts on natural assemblages. Ideally, the potentially impacted site and multiple reference sites are sampled through time, before and after the impact. Often, however, the lack of information regarding the potential overall impact, the lack of knowledge about the environment in many regions worldwide, budgets constraints and the increasing dimensions of human activities compromise the reliability of the impact assessment. We evaluated the impact, if any, and its extent of a nuclear power plant effluent on sessile epibiota assemblages using a suitable and feasible sampling design with no ‘before’ data and budget and logistic constraints. Assemblages were sampled at multiple times and at increasing distances from the point of the discharge of the effluent. There was a clear and localized effect of the power plant effluent (up to 100 m from the point of the discharge). However, depending on the time of the year, the impact reaches up to 600 m. We found a significantly lower richness of taxa in the Effluent site when compared to other sites. Furthermore, at all times, the variability of assemblages near the discharge was also smaller than in other sites. Although the sampling design used here (in particular the number of replicates) did not allow an unambiguously evaluation of the full extent of the impact in relation to its intensity and temporal variability, the multiple temporal and spatial scales used allowed the detection of some differences in the intensity of the impact, depending on the time of sampling. Our findings greatly contribute to increase the knowledge on the effects of multiple stressors caused by the effluent of a power plant and also have important implications for management strategies and conservation ecology, in general.

## Introduction

Human activities are causing rapid and substantial changes to Earth’s ecosystems [1]. Most of these changes occur before knowledge about the environment is generated. There are several sampling designs and statistical approaches to assess impacts on natural assemblages and/or systems [2], [3]. One of the most powerful ways to detect environmental impacts is the Beyond-BACI approach, where the potentially impacted site and multiple references sites are sampled through time, before and after the impact [3], [4]. Often, however, there is no environmental data available prior to the establishment of the possible source of impact, even in non-accidental impacts such as sewage or industrial effluents. In such cases, despite some limitations in establishing cause–effect relationships between human disturbances and responses of populations and/or assemblages, it is still possible to detect impacts, if any. Similar analyses, to what is advocated by the Beyond-BACI approach [5] are then usually applied considering multiple control sites and sampling across multiple temporal scales, limited to ‘after’ times only [6], [7].

Assessing impacts involves another problem: the lack of information or uncertainty about the extent of the affected area. In these cases, specific gradient designs with multiple temporal samplings, at several spatial scales, are essential because they allow distinction between natural temporal changes and those due to the impact [8], [9]. They also enable detection of changes in the variability within sites, which may be affected by impacts [3], [10].

Despite of the many publications available describing how to assess impacts [3], [4], [11]–[14], poorly designed studies are still being done and impacts are still being erroneously or incompletely assessed (as reviewed by [15]). This is particularly true for studies that evaluate the effects of different types of effluents on assemblages (but see e.g. [6], [9]).

Many coastal industries and power plants use sea-water in their cooling system and discharge this heated effluent into the sea, raising sea-water temperature in the vicinity of the discharge [16]–[18]. The effects of heated effluents seem to be more serious in tropical areas, especially in summer, when temperature of sea-water is near the upper tolerance limits of most marine organisms [19]. For example, in tropical or sub-tropical areas like Florida, India and Brazil, the increase of a few degrees in the temperature of sea-water caused by the effluents of power plants, decreased the biomass and richness of many benthic organisms [17], [20], [21]. Chlorine, which is deleterious for most biota, is usually added in the cooling systems to prevent fouling. In high concentrations, the chlorine released in the effluents may cause mortality and reduction in physiological activities of some benthic organisms [22], [23]. Also, the high turbulence and flow in the vicinity of the discharge can interfere in the settlement of many invertebrate larvae [24].

The aim of this study was to assess the impact, if any, of multiple stressors caused by the effluent of the Brazilian nuclear power plant (Central Nuclear Almirante Alvaro Alberto - CNAAA) on sessile epibiota, using a sampling design with budget and logistic constraints. We considered only the overall impact of the effluent, i.e. we did not separate the effect(s) of each stressor. At the time this field study was done, only three published studies on the effects of this power plant were found available, all on bioaccumulation on algae [25]–[27]. Only one biological study, on zooplankton, was available prior to the construction and functioning of the power plant [28]. We also use the sampling design done here as a case study to discuss the importance of appropriate and feasible sampling designs and statistical approaches to assess impacts, if any, and their extent.

The general hypotheses tested were that the power plant effluent would have an effect on the assemblages, reducing the number of species and the abundance of sessile epibiota near the discharge area and that this effect would vary in time and space. Especifically, we hypothesised that the effects of the effluent would decrease with increasing distances from the discharge area, being greater immediately next to the effluent. The effects of the effluent were also hypothesized to be greater when the average sea-water temperature in the region naturally reaches greater values (see Description of Area).

## Materials and Methods

### Description of Area

**Figure 1:**
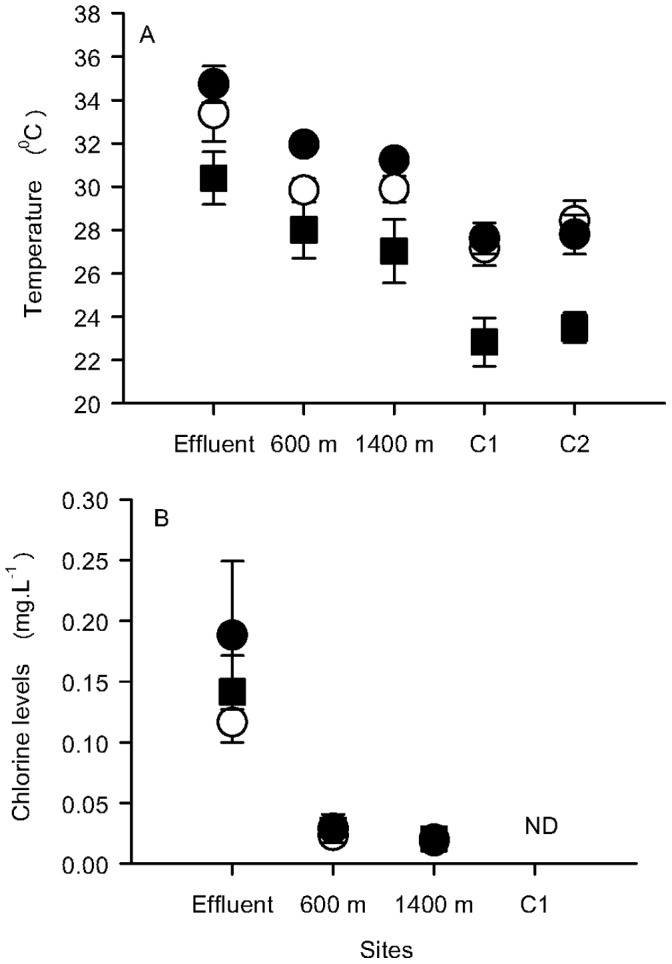
Mean values (±SE, n = 3) of sea-water temperature (A) and chlorine levels (B) found at different sites at Times 1 (square); 2 (white circle) and 3 (black circle). Measures were taken every month from July 2002 to April 2003. C1 = control 1 at the intake area; C2 = control 2– located at the East side of Brandão Island. ND = Not detected.

The CNAAA nuclear power plant discharge area (23° 07′S and 44° 26′ W) is located at Piraquara de Fora Inlet, in Ilha Grande Bay, on the southern coast of Rio de Janeiro state, Brazil. The power plant has been operating since 1985 and, at the time of this study, it consisted of two ^135^U pressurised water reactors producing around 1966 MW. The cooling system demands 120 m^3^.s^−1^ of sea-water and the effluent discharge causes an increase in the water flow creating a current of 30 cm.s^−1^ near the discharge point. Chlorine is added in a concentration of 1 mg.L^−1^ at the heat exchangers. At the discharge area, the mean water temperature and chlorine level are 32°C and 0.04 mg.L^−1^, respectively. The thermal plume forms with approximately 2 m depth. It spreads around the inlet and its extent varies depending on the capacity to which the power plant is operating and the tide and winds that constantly drive the plume for both South and North sides of the inlet [29]. Most areas of the bay have a mean water temperature of 27°C and undetectable levels of chlorine [29]. Temperature and chlorine levels were measured using a thermometer and a Merck chlorine kit, model Aquaquant 1,14434 with a precision range from 0.01 and 0.3 mg.L^−1^, respectively. These measures were taken every month for 10 months, from July 2002 to April 2003, at 0.5 m depth. The area immediately near the discharge had the greatest values of temperature of sea-water, with minimum values of 28°C in September, reaching up to 36°C in summer (i.e January and February), followed by the sites 600 m away from the discharge, which had temperatures of 27°C and 32°C in winter (i.e. July) and summer, respectively. The controls (C1 and C2) had the smallest temperatures registered for all sampled sites, with minimum of 22°C in winter and maximum values of 29°C in summer. Chlorine levels showed a marked decrease with increasing distances from the discharge area, (Figure 1), with the greatest values detected at the Effluent site, reaching up to 0.3 mg.L^−1^ in April 2003.

**Figure 2:**
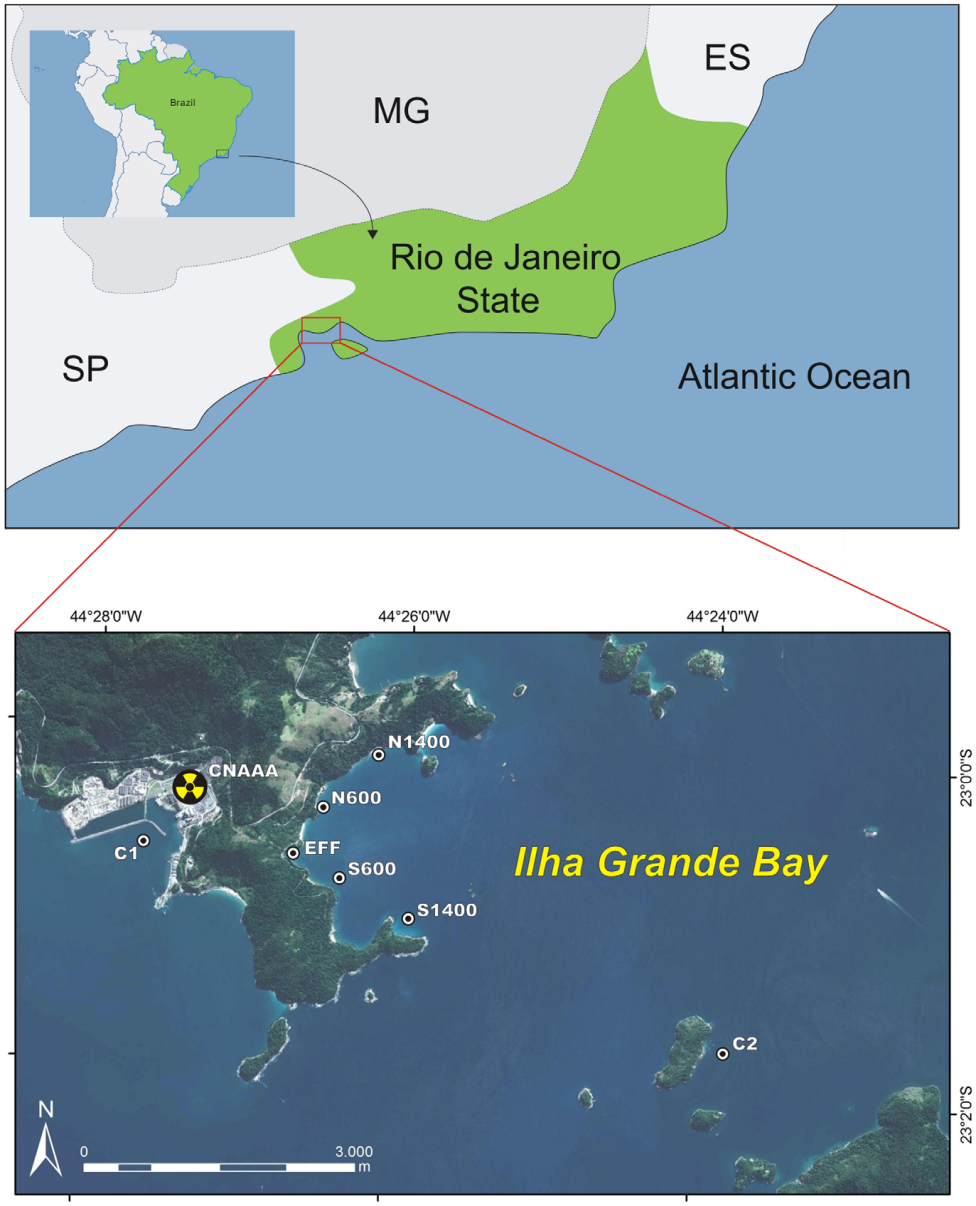
Map of the location of the CNAAA power plant and the sites sampled. Effluent (Eff) –100 m far from the discharge point of the power plant; 600 m sites (N600 and S600) – one on each side of the bay, 600 m far from the discharge point; 1400 m sites (N1400 and S1400) – one on each side of the bay, 1400 m far from the discharge point; Control 1 (C1) – the intake area of the cooling system, control area; Control 2 (C2) – located on the East side of Brandão Island, control area.

### Experimental Design

To determine the effects of the power plant’s effluent on sessile epibiota – if any – and its spatial extent, we used a sampling design with different spatial scales at multiple times. Seven sites were sampled, with increasing distances from the point of the discharge: Effluent (Eff) –100 m from the power plant discharge point and sites located at North and South of the discharge point located at 600 m (N600 and S600, respectively) and at 1400 m (N1400 and S1400, respectively). Two control sites were also sampled: Control 1 (C1) – located at the intake area of the power plant cooling system, approximately 3 km opposite to the area of the effluent discharge; Control 2 (C2) – located at the East side of Brandão Island, approximately 3 km away from the effluent discharge point (Figure 2). The control sites have the same natural type and similar slope of the susbtrata (i.e. vertical granite rocky shores) found on areas inside and around the Piraquara de Fora Inlet. These sites also present sessile assemblages similar to those commonly found in Ilha Grande Bay, except at localized disturbed areas (personal observation). No specific permits were required for the described field studies because the study did not involve destructive sampling on natural rocky shores nor any endangered or protected species.

Four independent granite panels of 20×20 cm were placed vertically at 0.5 m depth in each site. Panels were submerged for 3 months, being replaced at the end of this period. The replacement was repeated 4 subsequent times (T1, T2, T3 and T4) from July 2002 to June 2003. Panels were sorted under a dissecting microscope and the percentage cover of sessile organisms was estimated using 100 regularly spaced points in a grid. Micro-algae and protozoa that covered the panels were described as biofilm. Although most taxa were identified to genus or species for a biological inventory (see Tables S1 and S2), they were combined into categories (e.g. barnacles, biofilm, ascidians and bryozoans) for statistical analyses.

### Statistical Analyses

Asymmetrical univariate analyses of variance (ANOVA) were done to compare the total number of taxa and the percentage cover of the most abundant category (biofilm) among assemblages at different sites and times. Similarly, asymmetrical multivariate analyses of variance (PERMANOVA) were done to compare the structure of assemblages among sites at different times. The factors included in the analyses were: Distance, fixed; Sites, random and nested in Distance and Time, random and orthogonal. *A priori* contrasts were done to compare the relevant distances, i.e. because not all distances were to be compared among themselves, such as 600 m vs 1400 m, the distances to be compared were chosen *a priori* of the analyses (e.g. Eff *vs.* 600 m; Eff *vs.* Controls, etc.). Therefore, although the factor Distance has 4 levels in general (i.e. 100 m, 600 m, 1400 m and controls with >3000 m), in the *a priori* contrasts, this factor has only 2 levels (respective to the sites chosen to be compared, e.g. Eff vs Controls; see Tables in Results for further details of the analyses). When necessary, tests were done *a posteriori* of analyses of variance to separate significant means.

Due to the loss of several replicates at Times 3 and 4 at one of the control sites (C1), the analyses done with these sites only included Times 1 and 2, to avoid unbalanced analyses. The comparisons among the other distances (i.e. Eff vs 600 m and Eff vs 1400 m) included all the 4 sampling times (i.e. T1, T2, T3 and T4).

**Table 1:** Asymmetrical univariate analyses of variance of the number of taxa on panels submerged in different sites in all sampling times.

		Eff vs. 600 m		Eff vs 1400 m		Eff vs. Controls	
Source of variation	df	MS	F	MS	F	MS	F
Distance	3	401	11**	401	11**	184	2.7 ns
Distance	1	1014	110.0**	919	20.0*	391	23.8*
Time+	3	22	1.3 ns	22	1.3 ns	45	2.4 ns
Site (Di)	3	10	0.6 ns	10	0.6 ns	17.7	1.0 ns
Site (Di)	1	1	<0.0 ns	23	0.8 ns	8	1.0 ns
Di X Ti+	9	29	1.7 ns	29	1.7 ns	57.4	3.1 ns
Di X Ti+	3	9	0.9 ns	25	0.9 ns	9	1.2 ns
Si(Di) X Ti+	7	17	3.4*	17	3.4*	18.5	3.6*
Si(Di) X Ti+	3	10	1.9 ns	27	9.0**	8	2.7 ns
Residual+	78	5		5		5	

*n* = 4. The whole analyses is shown as well as the *a priori* contrast (i.e. the analyses where the distances to be compared were chosen *a priori*).

ns = not significant;

* = *p*<0.05;

** = *p*<0.01.

Eff  =  Effluent; 600 m and 1400 m = sites 600 and 1400 m away from the effluent, respectively. Factor 1: Distance (Di), fixed, with 2 levels; Factor 2: Sites (Si), random, nested in Distance; Factor 3: Time (Ti), random, with 4 levels (except in comparisons with the Control sites, where there were only 2 levels).

+ Because the analysis “effluent vs. controls” was only done for two times of sampling, the degrees of freedom of Time is 1, Di (Ti) and Lo(Di) X Ti are 3 and the residual is 42.

**Table 2:** Univariate analyses of variance of the number of taxa on panels submerged in different sites in all sampling times.

		600 m vs. Control		1400 m vs Controls	
Source of variation	df	MS	F	MS	F
Distance	3	184	2.7 ns	184	2.7 ns
Distance	1	11.3	0.4 ns	3	0.1 ns
Time	1	45	2.4 ns	45	2.4 ns
Si(Di)	3	17.7	1.0 ns	17.7	1.0 ns
Si(Di)	2	3.8	0.3 ns	27	1.5 ns
Di X Ti	3	57.4	3.1 ns	57.4	3.1 ns
Di X Ti	1	57.8	4.1 ns	162	9.2 ns
Si(Di) X Ti	3	18.5	3.6*	18.5	3.6*
Si(Di) X Ti	2	13.9	1.9 ns	18	4.6 ns
Residual	42	5		5	

*n* = 4. The whole analyses is shown as well as the *a priori* contrast (i.e. the analyses where the distances to be compared were chosen *a priori*).

ns = not significant;

* = *p*<0.05;

** = *p*<0.01.

600 m and 1400 m = sites 600 and 1400 m away from the effluent, respectively. Factor 1: Distance (Di), fixed, with 2 levels; Factor 2: Sites (Si), random, nested in Distance with 4 levels; Factor 3: Time (Ti), random, with 2 levels.

**Figure 3:**
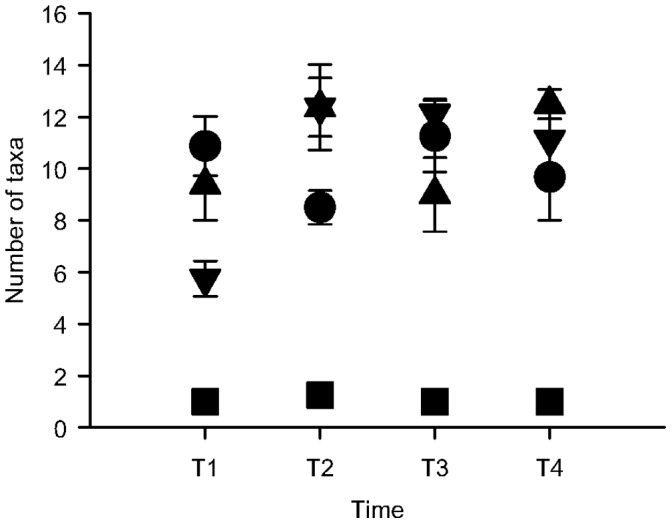
Number (± SE; *n* = 4 at the Effluent and and 8 at the other sites) of taxa found in each time at the Effluent site (squares), the sites 600 m (triangles) and 1400 m away from the effluent (inverted triangles) and at the control sites (circles). Note that in times T3 and T4, only one control (C2) was sampled due to losses of replicates.

The variability of assemblages within and among sites was calculated using PERMDIST. This was done calculating the deviations from the centroids in each site across all sampled times and within each time [30]. The average dissimilarities among and within sites were also calculated for each time of sampling, using Bray-Curtis dissimilarity index. Analyses were done using PRIMER multi-package 6.0. The univariate analyses of variance were also done using PRIMER to standardize the way the analyses were done (since no uni-variate statistical package can do asymmetrical analyses withouth partitioning the Sum of Squares; see e.g. [5], [13]). For the univariate analyses, Euclidean distance was used instead of Bray-Curtis (index used in the multivariate analyses).

## Results

Thirty-five taxa of macro-algae were recorded; 1 taxon at Effluent site, 20 at N600, 19 at S600, 16 at N1400, 21 at S1400, 9 at C1 and 12 at C2 site (Table S1). A total of 51 taxa of invertebrates were recorded, 1 taxon at Effluent site, 31 at N600, 20 at S600, 29 at N1400, 29 at S1400, 24 at C1 and 27 at C2 site (Table S2). No macroscopic taxon was found at all studied sites. The anemone *Haliplanella* was the only taxon exclusively found at the two control sites, whereas the Chlorophyta *Boodleopsis vaucherioidea* was just recorded at the two 600 m sites. No particular taxon was exclusively recorded at both 1400 m sites. Sabellidae (Polychaeta), the category Amphipods tubes and the sponge *Scopalina ruetzieri* were found at all sites at 600 m and 1400 m, however, these taxa were not found at the control sites (Tables S1 and S2).

Except for the Effluent site, the number of taxa within and among sites varied in time (Figure 3). At all times, the number of taxa was significantly smaller at the Effluent than at all the other studied sites (*p*<0.05, for values of F, please refer to Table 1). There were no significant differences among the 600 m and 1400 m sites with the controls (p>0.05; for values of F, please refer to Table 2).

**Figure 4:**
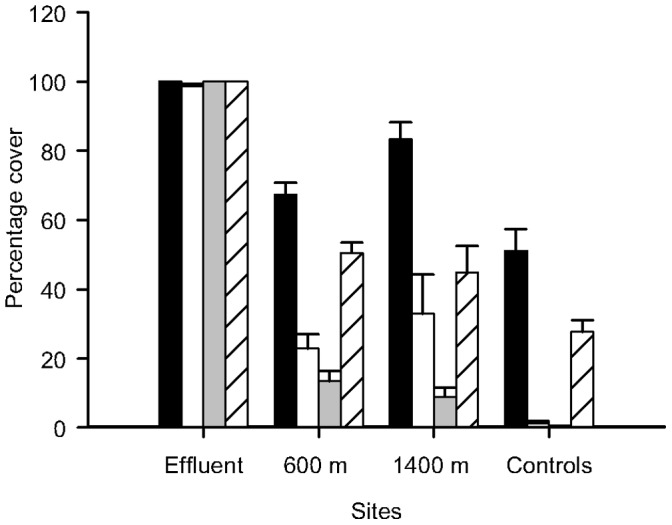
Percentage cover (±SE; *n* = 4 at the Effluent and 8 at the other sites) of Biofilm in the different sites, in each time of sampling. Note that in times T3 and T4, only one control (C2) was sampled due to losses of replicates. Black bars = T1; White bars = T2; Grey bars = T3; Striped bars = T4.

Covers of biofilm (which reflects a lack of recruitment of macro-organisms) showed great temporal and spatial variation (except at the Effluent), being greater at Time 1 than at other times of sampling (*p*<0.05; Figure 4). Biofilm cover was ∼100% at all sampling times at the Effluent site. Its cover was always significantly greater at the Effluent site when compared to the 600 and 1400 m sites (*p*<0.05; for values of *F*, please refer to Table 3). Control sites showed smaller values of cover of biofilm (Figure 4); however, statistical analyses showed no significant differences among the controls and the other sites, including the Effluent (*p*>0.05; for values of F please refer to Tables 3 and 4, please note that only Times 1 and 2 were analysed). The average dissimilarity among and within sites showed, however, a great difference of cover of biofilm among the Effluent and the control sites, with a dissimilarity among sites of approximately 98% at Time 2 (Table 5).

**Table 3:** Asymmetrical univariate analyses of variance of the percentage cover of biofilm on panels submerged in different sites in all sampling times.

		Eff vs. 600 m		Eff vs 1400 m		Eff vs. Controls	
Source of variation	df	MS	F	MS	F	MS	F
Distance	3	4.2	17.0**	4.2	17.0**	2.1	8.6**
Distance	1	8.5	47.0**	7.5	12.5**	6.2	11.6 ns
Time+	3	0.2	3.5 ns	0.4	5.1 ns	0.9	19.5 ns
Sites (Di)	3	<0.0	1.3 ns	<0.0	1.3 ns	<0.0	0.67 ns
Sites (Di)	1	<0.0	<0.0 ns	0.2	2.8 ns	<0.0	1 ns
Di X Ti+	9	0.2	2.3 ns	0.2	2.3 ns	0.2	1.4 ns
Di X Ti+	3	0.2	2.7 ns	0.4	4.6 ns	0.5	11.2 ns
Si(Di) X Ti+	7	<0.0	4.7**	<0.0	4.7**	0.1	6.4**
Si(Di) X Ti+	3	<0.0	10.7**	<0.0	6.8**	<0.0	5.2 *
Residual+	78	<0.0		<0.0		<0.0	

*n* = 4. The whole analyses is shown as well as the *a priori* contrast (i.e. the analyses where the distances to be compared were chosen *a priori*).

ns = not significant;

* = *p*<0.05;

** = *p*<0.01. Eff = Effluent; 600 m and 1400 m = sites 600 and 1400 m away from the effluent, respectively. Factor 1: Distance (Di), fixed, with 2 levels; Factor 2: Sites (Si), random, nested in Distance; Factor 3: Time (Ti), random, with 4 levels (except in comparisons with the Control sites, where there were only 2 levels). Data were arc-sin transformed.

+ Because the analysis “effluent vs. controls” was only done for two times of sampling, the degrees of freedom of Time is 1, Di (Ti) and Lo(Di) X Ti are 3 and the residual is 42.

**Table 4:** Univariate analyses of variance of the percentage cover of biofilm on panels submerged in different sites in all sampling times.

		600 m vs. Control		1400 m vs Controls	
Source of variation	df	MS	F	MS	F
Distance	3	2.1	8.6**	2.1	8.6**
Distance	1	0.7	5.8 ns	1.7	13.6 ns
Time	1	2.8	29.1*	3.5	30.7*
Si(Di)	3	<0.0	0.67 ns	<0.0	0.67 ns
Si(Di)	2	<0.0	0.4 ns	0.1	1.0 ns
Di X Ti	3	0.2	1.4 ns	0.2	1.4 ns
Di X Ti	1	<0.0	1.0 ns	<0.0	0.1 ns
Si(Di) X Ti	3	0.1	6.4**	0.1	6.4**
Si(Di) X Ti	2	<0.0	8.3**	0.1	4.1*
Residual	42	<0.0		<0.0	

*n* = 4. The whole analyses is shown as well as the *a priori* contrast (i.e. the analyses where the distances to be compared were chosen *a priori*).

ns = not significant;

* = *p*<0.05;

** = *p*<0.01.

600 m and 1400 m = sites 600 and 1400 m away from the effluent, respectively. N and S are sites in different sides of the bay (see Methods). Factor 1: Distance (Di), fixed, with 2 levels; Factor 2: Sites (Si), random, nested in Distance with 4 levels; Factor 3: Time (Ti), random, with 2 levels. Data were arc-sin transformed.

**Table 5:** Average Euclidean distance, considering only covers of biofilm, within the sites sampled at the discharge point (Effluent), 600 and 1400 m away from the discharge areas (600 m and 1400 m, respectively) and among control sites (C1 and C2), in each time.

Sites	Times	Effluent	600 m	1400 m	Controls
Within	T1	0.0	17.5	20.3	18.8
	T2	1.3	10.8	34.9	1.3
Among Controls	T1	48.9	21.3	25.0	
	T2	97.4	21.6	31.6	

**Figure 5:**
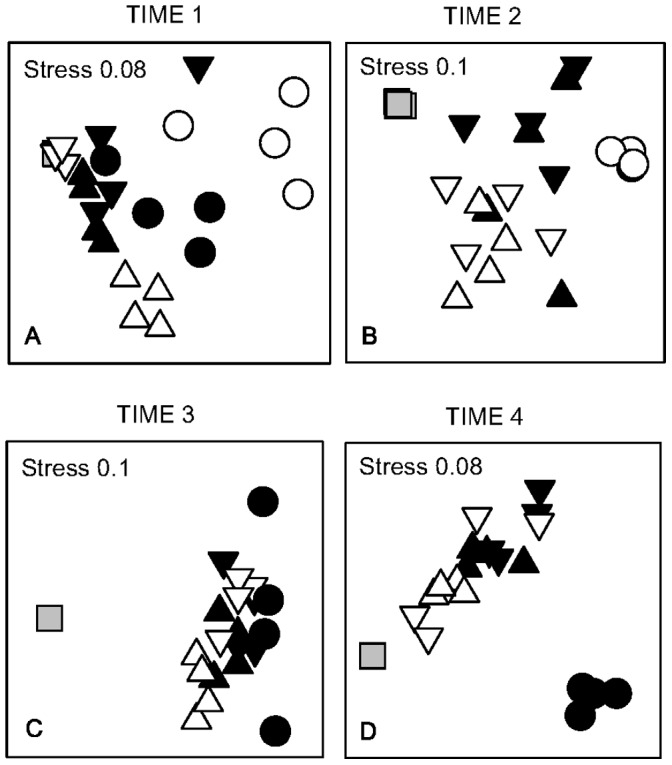
nMDS of assemblages on panels submerged for 3 months at the effluent site (squares), 600 m away from the effluent (triangles), 1400 m away from the effluent (inverted triangles) and controls (circles) in Time 1 (A), Time 2 (B), Time 3 (C) and Time 4 (D). Full symbols are to show sites on the same side of the bay (i.e. N or S) and to differentiate both controls (filled circles are control 1 - the intake, and empty circles are control 2). *n* = 4; Note that in times 3 and 4, only one control site (C2) was sampled due to losses of replicates.

Considering the whole assemblages, there was a great variability among sites and times (shown by the interaction Si (Di) X Ti; Tables 6 and 7). Despite of this variability, there was a clear difference among the Effluent site and the 600 m and 1400 m sites (Distance - *p*<0.05; for values of *F*, please refer to Table 6; Figure 5). The comparison between the Effluent and the control sites showed a significant interaction between Distance and Time (*p*<0.05; for values of *F*, please refer to Table 6), with the Effuent differing from the controls at Time 2 (*a posteriori* tests, Table 6). The 600 m sites significantly differed from the controls, but, similarly to the Effluent site, this difference was only significant at one of the two times analysed (i.e. Time 2; *p*<0.05; Table 7).

**Table 6:** Asymmetrical multivariate analyses on the structure of assemblages in different sites in all sampling times.

		Eff vs. 600 m		Eff vs 1400 m		Eff vs. Controls	
a) Source of variation	df	MS	F	MS	F	MS	F
Distance	3	18637	5.4**	18637	5.4**		
Distance	1	33903	9.2*	29652	11.5**	22755	4.1 ns
Time+	3	8178	6.5**	8178	6.5**	13272	6.9*
Sites (Di)	3	1463	1.2 ns	1463	1.2 ns		
Sites (Di)	1	2687	2.8 ns	968	0.6 ns	733	1.0 ns
Di X Ti	9	2291	1.8*	2291	1.8*		
Di X Ti+	3	1092	1.1 ns	1760	1.1 ns	4992	6.8*
Si(Di) X Ti+	7	1242	3.8**	1242	3.8**		
Si(Di) X Ti+	3	974	5.3**	1678	5.0**	733	3.5*
Residual+	78	323		323		342	
b) Post-hoc tests							
Time 1						Eff = Controls	
Time 2						Eff ≠ Controls	

*n* = 4. The whole analyses is shown as well as the *a priori* contrast (i.e. the analyses where the distances to be compared were chosen *a priori*).

ns = not significant;

* = *p*<0.05;

** = *p*<0.01.

Eff = Effluent; 600 m and 1400 m = sites 600 and 1400 m away from the effluent, respectively. Factor 1: Distance (Di), fixed, with 2 levels; Factor 2: Sites (Si), random, nested in Distance; Factor 3: Time (Ti), random, with 4 levels (except in comparisons with the Control sites, where there were only 2 levels). Data were squared-root transformed.

+ Because the analysis “effluent vs. controls” was only done for two times of sampling, the degrees of freedom of Time is 1, Di (Ti) and Lo(Di) X Ti are 3 and the residual is 42.

**Table 7:** Multivariate analyses on the structure of assemblages in different sites in all sampling times.

		600 m vs. Control		1400 m vs Controls	
a) Source of variation	df	MS	F	MS	F
Distance	3	10979	3.1*	10979	3.1*
Distance	1	12984	2.1 ns	8600	2.0 ns
Time	1	13272	7.0 *	13272	7.0*
Sites (Di)	3	1317	0.7 ns	1317	0.7 ns
Sites (Di)	2	1563	1.7 ns	778	0.3 ns
Di X Ti	3	2872	1.5 ns	2872	1.5 ns
Di X Ti	1	5104	5.6*	4614	2.0 ns
Si(Di) X Ti	3	1901	5.6**	1901	5.6**
Si(Di) X Ti	2	909	2.8*	2310	5.5**
Residual	42	342			
b) Post-hoc tests					
Time 1		600 m = Controls			
Time 2		600 m ≠ Controls			

*n* = 4 The whole analyses is shown as well as the *a priori* contrast (i.e. the analyses where the distances to be compared were chosen *a priori*).

ns = not significant;

* = *p*<0.05;

** = *p*<0.01.

600 m and 1400 m = sites 600 and 1400 m away from the effluent, respectively. Factor 1: Distance (Di), fixed, with 2 levels; Factor 2: Sites (Si), random, nested in Distance with 4 levels; Factor 3: Time (Ti), random, with 2 levels. Data were squared-root transformed.

In contrast, no significant differences were found between the controls and the sites 1400 m away from the discharge area in any of the analysed times.

There was a great variation within sites at Time 1, except at the Effluent site. At this time, the average dissimilarities within the 1400 m sites were greater than the dissimilarities among them and the Effluent site (23.3 and 16.6, respectively; Table 8). In contrast, the control sites (C1 and C2) showed ∼50% dissimilarity from all other sites, forming a distinct group (Table 8; Figure 5).

**Figure 6:**
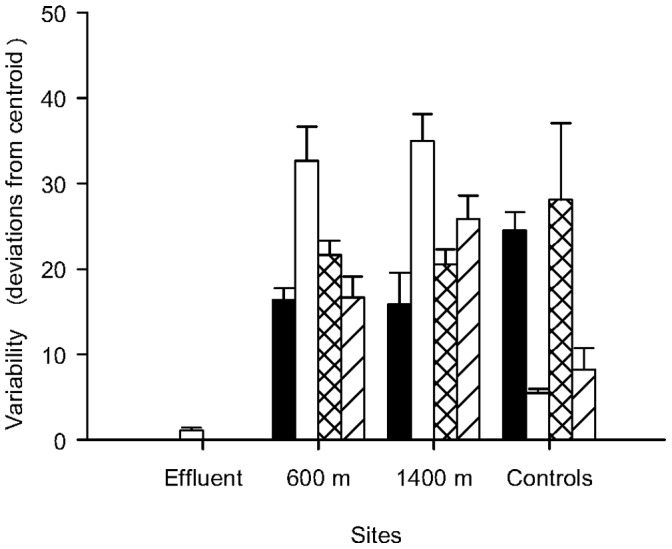
Variability in the assemblages within the Effluent site, the sites 600 m and 1400 m away from the effluent and within the controls. The deviations from the centroids in each site were calculated within each time. Black bars = T1; White bars = T2; Crossed bars = T3; Striped bars = T4. *n* = 4 at the Effuent and 8 at the other sites; Note that in Time 3 and 4, only one control site (C2) was analyzed due to losses of replicates.

The Effluent site had the smallest variability compared to all other sites, at all times. The variability of the assemblages varied greatly with time in all sites, except the Effluent (Figure 6).

## Discussion

Management actions, restoration efforts and even court penalties are, many times, a result of scientific information on detection of impacts and their consequences to the environment. Thus, the difficulty, or even inability, to detect an impact may have serious ecological and social consequences since contamination of natural systems, loss of diversity and ecosystem functions and benefits might be occurring with no legal penalties.

Suitable experiments, using appropriate designs and relevant spatial scales are necessary to properly assess impacts. In this study, a clear and localized effect of the power plant effluent – up to 100 m from the point of the discharge (i.e. Effluent site) – was found on the sessile epibiota. This was evident by differences concerning the total number of species, the biofilm cover and the variability of assemblages. The most obvious effect of the impact was the lack of macro-organisms on the Effluent site when compared to other parts of the bay, which could be due to impacts on settlement and/or post-settlement processes.

Despite the great variability found within sites (except for the Effluent site) and between times of sampling, the analyses of variance (specifically the multivariate ones) clearly showed a localized effect (up to 100 m from the point of the discharge) of the effluent on the sessile epibiota. Furthermore, it also showed that the impact can sometimes reaches up to 600 m of the discharge point, as demonstrated by the analyses at one of the four sampling times of this study.

The stressors of the nuclear power plant discharge (temperature, chlorine and flow) are recognized factors influencing the survival of invertebrate and macro-algae. The influence of temperature on distribution and abundance of several organisms is probably the most well-known of them [31], [32], [33] and one of the factors of main concern on the effects of climate change on natural systems [34], [35]. Changes in the composition and abundance of many species of algae and invertebrates, in California, have been attributed to the effect of an increase in sea-water temperature due to a heated power plant outfall [11], [36]. Similarly, near the effluent of a power plant in Florida, a 50% reduction of molluscan and crustacean taxa was observed at 33°C and a 75% reduction at 37°C [20]. In the effluent area of a nuclear power plant in India, almost the entire epifauna and macro-flora were eliminated at 37°C [17]. At the Effluent site studied here, temperature of sea-water reached up to 36°C, which could be one of the main factors for the lack of macro-organisms found at the site.

Chlorine has also been shown to have negative effects on many organisms [37], [38]. Concentrations of this contaminant similar to those found at the Efluent site (i.e. 0.1 mg.L^−1^) have been shown to decrease recruitment of bivalves [23]. Furthermore, the effluent studied here caused an increase in the flow of water in the area near the discharge point, creating a current of 30 cm.s^−1^ (data supplied by CNAAA). The flow of water may affect settlement of organisms by i) exerting hydrodynamic forces on settling propagules, ii) providing a settlement cue that induces active behaviour of motile propagules or iii) it may act to mediate various settlement cues [e.g. influencing sedimentation and chemical cues; 24]. High velocity water flow, such as those found near the power plant discharge point, can therefore reduce settlement of many species of organisms [24].

**Table 8:** Average dissimilarities among and within the sites sampled at the discharge point (Effluent), 600 and 1400 m away from the discharge areas (600 m and 1400 m, respectively) and control sites (C1 and C2), in each time.

Places	Times	Effluent	600 m	1400 m	Controls
Effluent	T1	0.00			
	T2	1.89			
	T3	0.00			
	T4	0.00			
600 m	T1	32.63	23.89		
	T2	75.79	49.29		
	T3	86.50	31.86		
	T4	49.50	25.21		
1400 m	T1	16.63	29.66	23.25	
	T2	66.00	51.58	52.57	
	T3	91.13	41.77	30.04	
	T4	55.13	35.61	38.36	
Controls	T1	48.90	41.42	44.15	36.68
	T2	97.92	72.47	63.03	7.68
	T3*	99.75	55.63	43.31	49.33
	T4*	72.33	61.07	61.91	14.39

* Note that at times 3 and 4, only one control was sampled (C2).

The multiple stressors of the power plant discharge studied here caused an impact on the sessile benthic assemblages at 100 m away from the discharge area (Effluent site). This impact varied in intensity depending on the time of the year, reaching up to 600 m at Time 2. Although at Time 1 there were no significant differences in the assemblages among the Effluent and the controls, this is probably due to the number of replicates used here (n = 4) and the great variation found within the assemblages at the control sites at this time. The number of taxa recorded in each site and the average dissimilarities among sites do suggest, however, a clear impact at the Effluent site at all times analysed. Furthermore, at all times, the variability within the Effluent site was also smaller than in other sites (i.e. 600 m, 1400 m and control sites). Environmental disturbances can affect variance instead of the means of biological variables, i.e. temporal and spatial variability inherent to natural assemblages may be affected by impacts [3], [10]. Similarly to the Effluent site, there were significant differences in the structure of assemblages among the sites 600 m away from the discharge area and the control sites at only one of the two analysed times.

No significant differences between the controls and 1400 m sites were found. This could be due to an actual lack of effect of the effluent at this distance or simply due to a lack of power of the analyses. Considering that, at those sites, sea-water temperature and chlorine levels were greater than at the control sites and that the qualitative and descriptive analyses (such as the nMDS and the composition of species in each site) suggest some differences among these sites and the controls, it is possible that these differences were not detected by the analyses of variance due to the low number of replicates used. Furthermore, only two times of sampling were analysed in comparisons with the control sites (due to losses of replicates, instead of the 4 times analysed in comparisons with the other distances); and there was a great variation found within the assemblages at the controls sites (especially at Time 1). To establish, with some certainty, whether the (more subtle) effects, if any, of the effluent did actually reach greater distances than those found here (i.e. up to 600 m depending on the time of the year), a greater number of replicates and/or sampling times should have been used. In addition, although the sampling design used was appropriate for the hypotheses being tested (i.e. the effluent has an impact on assemblages and this impact varies in time and according to distance form the discharge), it does not allow determining changes in spatial variation of the impact. For that, the spatial scales at which sampling was done within the bay with the outfall would need to be replicated at each of two control sites.

Budget and logistic constraints are, unfortunately, relevant in many situations regarding environmental impact assessments, especially in developing countries, making it very hard to apply what would be considered ‘ideal’ sampling designs. Extra-care should be taken in experimental designs that suffer budget and logistic constraints because, even with limited funding, parts of the sampling design cannot be eliminated or simplified without leading to an incomplete or erroneous environmental assessment. Low-budget studies that lack appropriate controls and replicates, may end up, in reality, costing more because they cannot determine whether an impact is, in fact, occurring or not. In this study, the strong impact of the power plant effluent on the sessile assemblages at 100 m of the discharge point (Effluent site) could probably have been detected even using simpler sampling designs. However, although the design used here (in particular the number of replicates) did not allow an unambiguously evaluation of the full extent of the impact caused by the power plant discharge in relation to its intensity and temporal variability, the multiple temporal and spatial scales used allowed the detection of some differences in its intensity and temporal variability.

Our findings have important implications for management strategies, not only of the Brazilian nuclear power plant, but also for management and conservation ecology in general. We emphasize that evidence-based knowledge is critical for building effective impact evaluation (considering its extent, intensity and spatial and temporal variation) to define monitoring strategies for basic or applied ecology.

## Supporting Information

Table S1 **Taxa of macro-algae found in each sampled site across all times.**
(DOC)

Table S2 **Taxa of invertebrates found in each sampled site across all times.**
(DOC)
