# Supplementary material for: How Much Is Too Little to Detect Impacts? A Case Study of a Nuclear Power Plant
Source: PLoS One. 2012 Oct 26;7(10):e47871. doi: 10.1371/journal.pone.0047871 (PMC3482239; doi:10.1371/journal.pone.0047871)
Supplement: Table S1 — Taxa of macro-algae found in each sampled site across all times. (DOC) [file pone.0047871.s001.doc]

Table S1. Taxa of macro-algae found in each sampled site across all times.

|  | EFF | N600 | S600 | N1400 | S1400 | C1 | C2 |
| --- | --- | --- | --- | --- | --- | --- | --- |
| Chlorophyta |  |  |  |  |  |  |  |
| *Boodleopsis vaucherioidea* |  | X | X |  |  |  |  |
| *Chaetomorpha minima* |  |  | X | X | X | X | X |
| *Cladophora* sp. |  | X |  |  |  |  |  |
| *Cladophora vagabunda* |  | X |  | X | X |  |  |
| *Derbesia tenuissima* |  |  | X |  | X | X |  |
| *Ulva chaetomorphoides* |  | X | X |  |  | X |  |
| *Ulva flexuosa* |  | X | X |  | X | X |  |
| Phaeophyta |  |  |  |  |  |  |  |
| *Colpomenia sinuosa* |  |  | X |  | X |  |  |
| *Feldmannia indica* |  |  |  |  | X | X |  |
| *Feldmannia irregularis* |  | X | X | X | X |  | X |
| *Hincksia mitchelliae* |  | X | X |  |  |  | X |
| *Sphacelaria rigidula* |  | X | X |  | X |  | X |
| RHODOPHYTA |  |  |  |  |  |  |  |
| *Acanthophora spicifera* |  | X | X | X | X |  | X |
| *Aglaothamnion boergesenii* |  | X | X | X | X | X |  |
| *Aglaothamnion felliponei* |  | X | X | X | X |  |  |
| *Aglaothamnion uruguayense* |  | X |  |  |  |  |  |
| *Amphiroa* sp. |  |  |  | X |  |  |  |
| *Antithamnion cruciatum* |  |  |  |  |  |  | X |
| *Asparagopsis taxiformis* |  | X | X | X | X |  | X |
| *Centroceras* sp. |  |  |  |  |  |  | X |
| *Ceramium comptum* |  | X |  |  | X |  |  |
| *Ceramium* sp. |  |  |  |  |  | X |  |
| *Champia compressa* |  |  |  | X |  |  |  |
| *Chondria atropurpurea* |  |  | X |  |  |  |  |
| *Dasya* sp. |  | X | X |  | X |  |  |
| *Gayliella* sp. | X | X | X | X | X |  | X |
| *Griffithsia schousboei* |  |  | X |  |  |  |  |
| *Herposiphonia secunda* |  |  |  | X |  |  |  |
| *Heterosiphonia crispella* |  |  |  |  | X |  |  |
| *Heterosiphonia gibbesii* |  |  |  | X |  |  |  |
| *Hypnea spinella* |  |  |  | X |  |  |  |
| *Jania capillacea* |  | X |  |  | X | X | X |
| *Neosiphonia howei* |  |  |  |  | X | X |  |
| *Polysiphonia subtilissima* |  | X | X | X | X |  | X |
| *Wrangelia argus* |  | X |  | X | X |  |  |
| Encrusting calcareous algae |  | X | X | X | X |  | X |
| TOTAL of algae | 1 | 20 | 19 | 16 | 21 | 9 | 12 |
