# Supplementary material for: How Much Is Too Little to Detect Impacts? A Case Study of a Nuclear Power Plant
Source: PLoS One. 2012 Oct 26;7(10):e47871. doi: 10.1371/journal.pone.0047871 (PMC3482239; doi:10.1371/journal.pone.0047871)
Supplement: Table S2 — Taxa of invertebrates found in each sampled site across all times. (DOC) [file pone.0047871.s002.doc]

Table S2. Taxa of invertebrates found in each sampled site across all times.

|  | EFF | N600 | S600 | N1400 | S1400 | C1 | C2 |
| --- | --- | --- | --- | --- | --- | --- | --- |
| Cnidaria |  |  |  |  |  |  |  |
| Actiniaria |  |  |  |  |  | X | X |
| *Haliplanella* sp. |  |  |  |  |  | X |  |
| Hydrozoa |  | X | X | X | X | X | X |
| Bryozoa |  |  |  |  |  |  |  |
| Encrusting bryozoan |  | X |  | X | X | X | X |
| Branching bryozoan |  | X |  | X |  | X | X |
| Stoloniferous bryozoan |  | X |  |  |  | X | X |
| Crustacea |  |  |  |  |  |  |  |
| Balanomorpha recruits | X |  |  |  |  |  |  |
| *Amphibalanus amphitrite* |  | X | X | X | X | X | X |
| *Amphibalanus eburneus* |  | X | X | X | X | X | X |
| *Amphibalanus improvisus* |  | X | X |  |  | X |  |
| *Amphibalanus reticulatus* |  | X | X | X | X | X | X |
| *Balanus trigonus* |  | X | X | X | X | X | X |
| *Chthamalus proteus* |  |  | X | X |  |  | X |
| *Fistulobalanus citerosum* |  |  |  |  | X |  |  |
| *Megabalanus coccopoma* |  | X | X | X | X | X | X |
| *Megabalanus tintinnabulum* |  | X | X | X | X | X | X |
| *Newmanella radiata* |  | X | X | X | X | X | X |
| *Tetraclita stalactifera* |  |  |  |  |  | X |  |
| Amphipods tube |  | X | X | X | X |  |  |
| Mollusca |  |  |  |  |  |  |  |
| *Isognomon bicolor* |  |  |  |  |  | X |  |
| *Musculus viator* |  | X |  | X | X | X | X |
| Ostreidae |  |  |  |  |  |  | X |
| *Perna perna* |  |  |  |  |  | X |  |
| *Petaloconchus* sp. |  |  |  |  |  |  | X |
| *Pinctada embricata* |  |  |  |  |  | X |  |
| *Pteria hirundo* |  | X |  | X | X | X | X |
| Polychaeta |  |  |  |  |  |  |  |
| Sabellidae |  | X | X | X | X |  |  |
| Serpulidae |  | X | X | X | X | X | X |
| Spionidae |  | X | X |  | X |  |  |
| Porifera |  |  |  |  |  |  |  |
| Calcarea |  | X |  |  |  |  | X |
| *Callyspongia* sp. |  | X |  |  | X |  |  |
| *Haliclona* sp. |  |  |  |  | X |  |  |
| *Mycale angulosa* |  |  |  |  | X |  |  |
| *Mycale microsigmatosa* |  |  |  |  |  |  | X |
| *Scopalina ruetzieri* | 0 | X | X | X | X |  |  |

Cont. of Table S1. Taxa of invertebrates found in each sampled site across all times.

|  | EFF | N600 | S600 | N1400 | S1400 | C1 | C2 |
| --- | --- | --- | --- | --- | --- | --- | --- |
| CHORDATA - Ascidiacea |  |  |  |  |  |  |  |
| Unidentified colonial ascidian |  | X |  | X |  |  |  |
| Botryllinae |  |  | X |  |  |  |  |
| *Botrylloides nigrum* |  | X | X | X | X |  | X |
| Didemnidae |  | X |  | X | X | X | X |
| *Didemnum ahu* |  | X |  | X |  | X | X |
| *Didemnum* sp. |  |  |  | X |  |  |  |
| *Diplosoma listerianum* |  | X | X | X | X |  | X |
| *Diplosoma* sp. |  | X | X | X | X |  |  |
| *Eusynstyela* sp. |  | X |  |  |  |  |  |
| *Herdmania pallida* |  |  | X | X | X |  | X |
| *Lissoclinum* sp. |  |  |  | X |  |  |  |
| *Phallusia nigra* |  |  |  | X | X | X | X |
| Polyzoinae |  | X |  |  | X |  |  |
| Styelidae |  |  |  | X | X |  |  |
| Styelinae |  | X |  |  |  |  |  |
| *Symplegma brakenhielmi* | 0 | X |  | X | X |  |  |
| Total of Invertebrates | 1 | 31 | 20 | 29 | 29 | 24 | 27 |
